# PCR-based reverse genetics strategy for bluetongue virus recovery

**DOI:** 10.1186/s12985-019-1261-2

**Published:** 2019-12-05

**Authors:** Qingyuan Xu, Jinying Ge, Maolin Li, Encheng Sun, Yawei Zhou, Yunze Guo, Donglai Wu, Zhigao Bu

**Affiliations:** 1 The Key Laboratory of Veterinary Public Health, Ministry of Agriculture, State Key Laboratory of Veterinary Biotechnology, Harbin Veterinary Research Institute, Chinese Academy of Agricultural Sciences, Harbin, 150001 People’s Republic of China; 2 College of Veterinary Medicine, Inner Mongolia Agricultural University, Hohhot, Inner Mongolia 010018 People’s Republic of China; 3 State Key Laboratory of Veterinary Biotechnology, Lanzhou Veterinary Research Institute, Chinese Academy of Agricultural Sciences, Lanzhou, 730030 People’s Republic of China

**Keywords:** Bluetongue virus, Reverse genetics, T7 RNA polymerase, Genome modification, Reassortment

## Abstract

**Background:**

Bluetongue virus (BTV), an emerging insect vector mediated pathogen affecting both wild ruminants and livestock, has a genome consisting of 10 linear double-stranded RNA genome segments. BTV has a severe economic impact on agriculture in many parts of the world. Current reverse genetics (RG) strategy to rescue BTV mainly rely on in vitro synthesis of RNA transcripts from cloned complimentary DNA (cDNA) corresponding to viral genome segments with the aid of helper plasmids. RNA synthesis is a laborious job which is further complicated with a need for expensive reagents and a meticulous operational procedure. Additionally, the target genes must be cloned into a specific vector to prepare templates for RNA transcription.

**Result:**

In this study, we have developed a PCR based BTV RG system with easy two-step transfection. Viable viruses were recovered following a first transfection with the seven helper plasmids and a second transfection with the 10 PCR products on the BSR cells. Further, recovered viruses were characterized with indirect immunofluorescence assays (IFA) and gene sequencing. And the proliferation properties of these viruses were also compared with wild type BTV. Interestingly, we have identified that viruses containing the segment 2 of the genome from reassortant BTV, grew slightly slower than the others.

**Conclusion:**

In this study, a convenient PCR based RG platform for BTV is established, and this strategy could be an effective alternative to the original available BTV rescue methods. Furthermore, this RG strategy is likely applicable for other Orbiviruses.

## Introduction

Bluetongue virus (BTV) is a species in the genus Orbivirus which is the largest of 15 genera within the family of Reoviridae. BTV is transmitted between mammalian hosts by certain biting midges (*Culicoides* spp.) and can infect all ruminant species [1]. The distribution of BTV is directly associated with the presence of competent vectors and their habitats, and this virus has been found on all continents except Antarctica according to the OIE report (http://www.oie.int/report2018). In fact, the global distribution of BTV was relatively stable at temperate and tropical latitudes between approximately 40–50°N and 35–40°S until 1998 [2, 3]. However, the distribution of BTV has profoundly changed with the invasion and spread of this disease throughout much of Europe [4]. A BTV serotype 8 outbreak in Europe indicated that the BTV epidemic range has extended to 53°N [5]. BTV, with 27 serotypes, is one of the most widespread animal pathogens and acts as an important representative of this class of large non-enveloped viruses [6]. BTV infection can cause considerable economic consequences due to both the disease itself and the resulting restrictions in international livestock trade.

BTV genome is composed of 10 linear segments of double-stranded RNA (dsRNA) encoding seven structural proteins (VP1–VP7) and four non-structural proteins (NS1, NS2, NS3/NS3a and NS4) [7–9]. BTV genome segments are classified from segment 1 to segment 10 in decreasing order of size. BTV has a layered structure, with the outer layer being separated before the remaining core particle enters the cytoplasm of the host cell [10]. The core particle which has transcriptionally active can synthesize and extrude multiple capped single-stranded mRNA copies of each viral genome segment into the host cell cytoplasm. The development of reverse genetics (RG) system of viruses is considered one of the most transformative technological advances in virology, having allowed for considerable progress to be made in understanding multiple aspects of virus biology and pathogenesis. However, the establishment of BTV RG is understood to be challenging, not only due to the complex genomic structure but also due to the lack of clarity regarding this virus’s replication and assembly process. Recent establishment of BTV infection process using transfected capped mRNAs into permissive cells by Polly Roy and her colleagues allowed the landmark progress in the field of BTV RG technology [10]. Henceforward, other RG strategies were developed, including transfected T7 polymerase derived mRNAs with or without assistant plasmid transfection [11, 12]. Recently, 10 plasmids BTV RG system has been developed [13]. Almost all of the existing BTV RG techniques need many plasmid constructions along with RNA synthesis to successfully recover the viruses.

Despite the great success of these technologies and variations to the plasmid-based approach having been developed, they all inevitably rely on a plasmid construction step and most methods need mRNA synthesis in vitro. An RG system which does not rely on RNA transcription in vitro and which could reduce the number of plasmid construction required, could speed up studies on understanding the significance of mutations in the viral genome for replication and/or modulation of virulence. In this report, PCR amplicons, instead of plasmids or mRNAs, are established as an efficient and viable alternative, when compared to the previous BTV RG systems.

## Material and method

### Cells culture

BHK-21 cells were cultured at 37 °C and 5% CO_2_ in a minimum essential medium, DMEM (Gibco) with 5% (v/v) fetal bovine serum (Excel) and antibiotics (100 U/mL penicillin G, 0.1 mg/mL streptomycin).

BSR cells, which stably expressed bacteriophage T7 RNA polymerase, were cultured in same culture medium and were conditioned with BHK-21 cells. 100 μg/mL of geneticin (G418) was added into minimum essential medium before performing the viral rescue to keep the stable expression of T7 RNA polymerase in the BSR cells.

### Helper plasmids and transfected PCR amplicons preparation

**Fig. 1:**
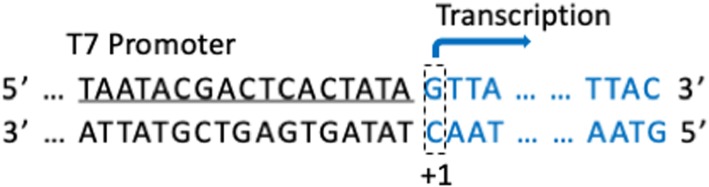
Schematic representation for PCR amplicon. Stably expressing T7 polymerase, and DNA with T7 polymerase promoter could be transcribed into RNA in the BSR cell. To prepare the template for RNA transcription to rescue BTV with RG system, the T7 polymerase promoter was introduced into the 5′ terminal of each gene by PCR method. The first nucleotide of BTV segments was located at transcriptional start site of T7 polymerase and the last nucleotide of PCR amplicon was the last one of BTV segments. This ensures the RNA synthesized with theses templates in the BSR are exactly same as corresponding original segments of BTV

The seven helper plasmids were constructed according to our previous report [14]. Briefly, the open reading frames of VP1, VP3, VP4, VP6, VP7, NS1 and NS2 of BTV-1 (strain SZ97/1) were cloned and inserted into pCI-neo vector, and the recombinant plasmids were designated as pCI-VP1, pCI-VP3, pCI-VP4, pCI-VP6, pCI-VP7, pCI-NS1 and pCI-NS2, respectively. These helper plasmids were purified with PureLink™ HiPure Plasmid Maxiprep Kit (Invitrogen) and the concentrations of the plasmids were identified with Micro UV spectrophotometer. The PCR amplicons of 10 segments of BTV-1 were amplified with specific primers (Table 1) from the templates of the T-vectors containing segment 1 to segment 10 genes of the BTV-1 (strain SZ97/1), which were maintained in our laboratory. The T7 polymerase promoter sequence was introduced into 5′ terminal of each gene with primers as shown in italic in Table 1. Segment 2 (JN255863) of BTV-2IT (L) was synthesized (GENERAL BIOSYSTEMS Co. Ltd), and T7 polymerase promoter was introduced at the 5′ end of it with specific primers (Table 1). The strategy to construct each amplicon is as shown in Fig. 1, T7 polymerase promoter was located upstream of each BTV segment, the first nucleotide of each BTV segment was designed at transcriptional start site, and there is no extra nucleotide at the 3′ end of each BTV segment in the amplicon. This ensures that the RNA sequence synthesized in BSR cells are exactly the same as the original segment of BTV. The PCR products were purified with PCR clean Kit (Axygen) according to the manufacturer’s instructions, and the concentration of the purified amplicons were identified with Micro UV spectrophotometer.

**Table 1:** Primers for BTV1 ten segments and BTV2 segment 2

Primer	Sequence (5′-3′)	Amplicon
S1–1-20-F	*TAATACGACTCACTATA*GTTAAAATGCAATGGTCGCA	BTV1 segment 1
S1–3944-3925-R	GTAAGTGTAATGCGGCGCGT	
S2–1-20-F	*TAATACGACTCACTAT*AGTTAAAATAGTAGCGCGATG	BTV1 segment 2
S2–2940-2921-R	GTAAGTCTAATAGTGCGCGG	
S3–1-20-F	*TAATACGACTCACTATA*GTTAAATTTCCGTAGCCATG	BTV1 segment 3
S3–2772-2753-R	GTAAGTGTGTTCCCGCTGCC	
S4–1-20-F	*TAATACGACTCACTATA*GTTAAAACATGCCTGAGCCA	BTV1 segment 4
S4–1981–1962-R	GTAAGTTGTACATGCCCCCC	
S5–1-20-F	*TAATACGACTCACTATA*GTTAAAAAAGTTCTCTAGTT	BTV1 segment 5
S5–1772–1753-R	GTAAGTTGAAAAGTTCTAGT	
S6–1-20-F	*TAATACGACTCACTATA*GTTAAAAAGTGCGCCCTTAG	BTV1 segment 6
S6–1635–1616-R	GTAAGTGTAAGTGCTTCCCG	
S7–1-20-F	*TAATACGACTCACTATA*GTTAAAAATCTATAGAGATG	BTV1 segment 7
S7–1156–1137-R	GTAAGTGTAATCTAAGAGAC	
S8–1-20-F	*TAATACGACTCACTATA*GTTAAAAAATCCTTGAGTCA	BTV1 segment 8
S8–1125–1106-R	GTAAGTGTAAAATCCCCCCC	
S9–1-20-F	*TAATACGACTCACTATA*GTTAAAAAATCGCATATGTC	BTV1 segment 9
S9–1049–1030-R	GTAAGTGTAAAATCGCCCTA	
S10–1-20-F	*TAATACGACTCACTATA*GTTAAAAAGTGTCGCTGCCA	BTV1 segment 10
S10–822-803	GTAAGTGTGTAGCGCCGCAT	
rS2–1-20-F	*TAATACGACTCACTATA*GTTAAAACAGGATCGCGATG	BTV2 segment 2
rS2–2943-2924-R	GTAAGTTGAACAGATCGCGG

### Generation of BTVs by RG using PCR amplicons and helper plasmids

**Fig. 2:**
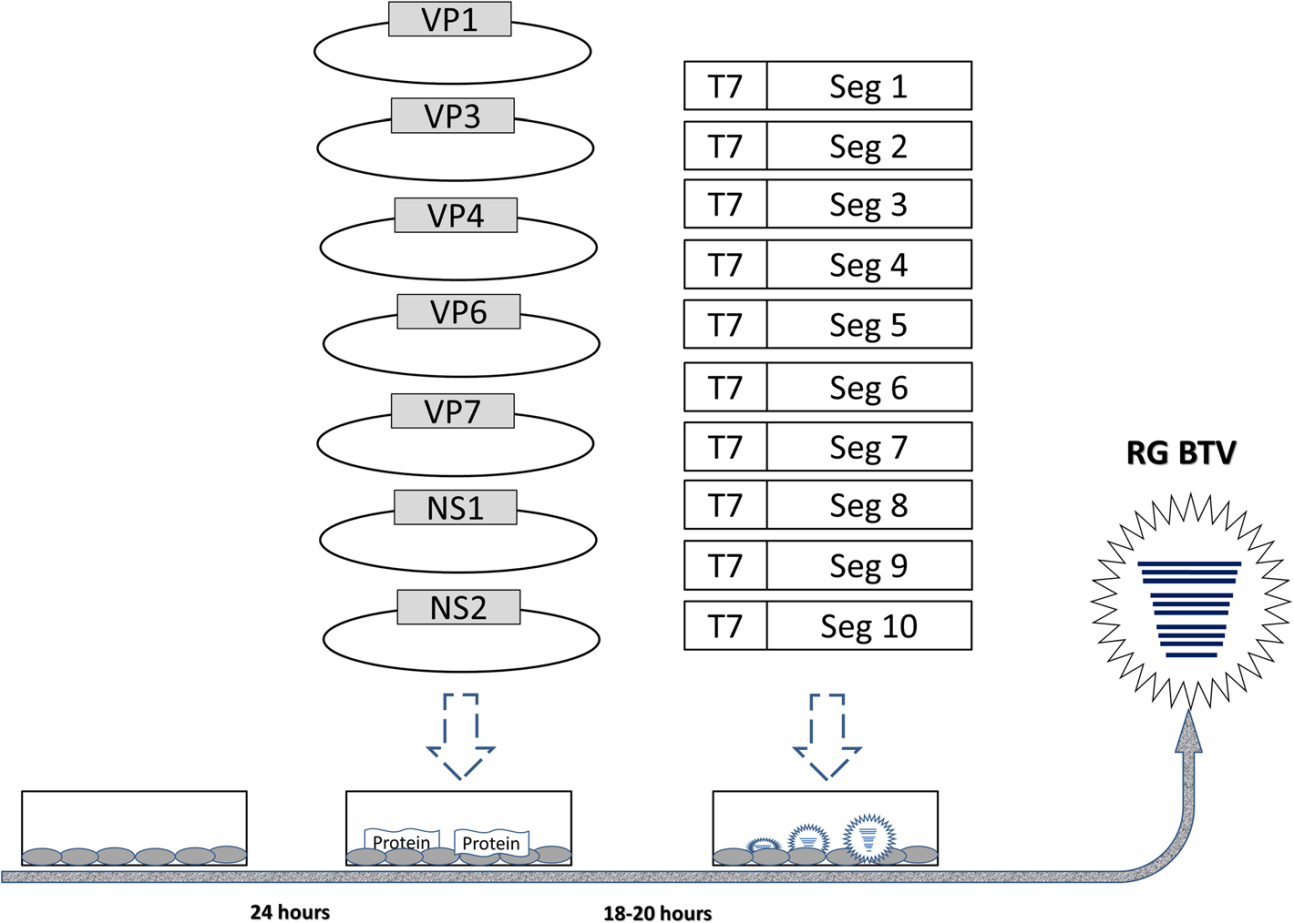
Diagrammatic sketch of PCR-based reverse genetics. BSR cells were seeded into 6-well tissue culture plates and when the cells reached 90% confluency, 3200 ng of seven helper plasmids were transfected into the BSR cells. The BTV proteins were expressed and necessary protein for BTV assembly and replication were prepared. Then 2000 ng of 10 PCR amplicons were transfected into BSR cells. BTV genome genes were produced by T7 polymerase in BSR cells. Then new BTV were recovered with these proteins and genome genes. In most case, the CPE could be observed 3–5 days after PCR amplicons transfection

BTV1 was rescued with two-step transfection protocol, transfection of helper plasmids is the first step followed by the transfection of PCR amplicons (Fig. 2). Briefly, BSR cells was seeded into wells of a 6-well tissue culture plate and cultured at 37 °C for 24 h. The following day, when the cells grew up to 90% confluency, helper plasmids were transfected into BSR cells with lipofectamine™ 3000 reagent (Invitrogen) according to the manufacturer’s instructions. Briefly, 7.5 μL of lipofectamine™ 3000 was mixed into 125 μL of OPTI-MEM, mixed well and designated as solution A. Helper plasmids were added to 125 μL of OPTI-MEM containing 4 μL of P3000 reagent, mixed well and designated as solution B. The helper plasmids dosages were as follows: 700 ng of pCI-VP1, 600 ng of pCI-VP3, 500 ng of pCI-VP4, 300 ng of pCI-VP6, 300 ng of pCI-VP7, 500 ng of pCI-NS1 and 300 ng of pCI-NS2. Solution A and solution B were mixed and incubated at room temperature for 15 min. The BSR cell culture medium was replaced with 2 mL of OPTI-MEM. Then 250 μL of solution A and solution B mixture were added onto the BSR cells. And these BSR cells were continued to be cultured at 37 °C in 5% CO_2_ cell incubator. About 20 h after the first transfection, the PCR products with the BTV segments were transfected into the same BSR cell, and this is the second transfection step. The transfection protocol of PCR amplicon was similar to the helper plasmid transfection except we replaced helper plasmids with PCR amplicons. The amplicons dosages of each segment were as follows: 400 ng of segment 1, 300 ng of segment 2, 300 ng of segment 3, 200 ng of segment 4, 200 ng of segment 5, 200 ng of segment 6, 100 ng of segment 7, 100 ng of segment 8, 100 ng of segment 9 and 100 ng of segment 10. The transfected cells were incubated in CO_2_ cell incubator until the marked cytopathic effect (CPE) was observed. Recombinant viruses were harvested and infected on BHK-21 cells for further identification. At the same time, mock transfection, helper plasmids only transfection and PCR products only transfection were also conducted as controls. In the mock transfection well, we did not add any helper plasmids or PCR products. When recombinant BTV1 which contained the segment 2 of BTV2IT(L) was rescued, the similar two-step transfection method was employed, except the segment 2 of BTV-1 (SZ97/1) was replaced with segment 2 of BTV2IT(L) in the second transfection step.

### Identification of rescued BTVs with immunofluorescence assay

BHK-21 cells grown on 12-well plates and the cells were infected with rescued BTV at a dose of 100 TCID_50_/well or or 100 μL of each control group supernatant, respectively. Forty-eight hours post infection, the cells were washed three times with precooled 0.01 M phosphate buffered saline (PBS) buffer and fixed in 4% paraformaldehyde for 30 min at 4 °C, then incubated with 0.1% Triton X-100 at 4 °C for 30 min. The cells were incubated with a primary anti-BTV VP7 antibody (designated as BTV-4H7) for 1 h at 37 °C [15]. The cells were further incubated with fluorescein isothiocyanate (FITC)-conjugated goat anti-mouse IgG Fc for 1 h at 37 °C. The cells were washed five times with PBST (PBS with 0.05% Tween-20) after each step. Finally, the cells were examined under a fluorescence microscope.

### Identification of reassortant viruses with PCR product sequencing

To identify reassortant BTV (which contained the Segment 2 of BTV2), the viral dsRNA was extracted from 250 μL of virus stocks with TRIzol™ LS reagent (Thermo) according to the manufacturer’s instruction. The cDNA was synthesized from the viral RNA and PCR amplification was performed using S2 segment-specific primer pairs for BTV-2IT(L) (Ls2–65-85-F: 5′-TTGGCATTCCGATTTATGGAC-3′ and Ls2–655-674-R: 5′-TTATCTGAAGTTGACGCTCT-3′). An AMV Reverse Transcriptase (TaKaRa) was applied for cDNA synthesis, 3 μL of RNA and 1 μL of primer Ls2–655-674-R were mixed and denatured at 95 °C for 5 min and immediately cooled on ice. RNase-free water of 5 μL, 4 μL of 5x AMV Buffer, 0.5 μL of RNase inhibitor (TaKaRa) and 1 μL of AMV was added into the mixture containing RNA and primer. Further, the samples were incubated at 25 °C for 10 min, 42 °C for 60 min and 70 °C for 5 min. The PCR reaction mixture contained 25 μL of PrimeSTAR HS (Premix), 1 μL of Ls2–65-85-F, 1 μL of Ls2–655-674-R, 4 μL of cDNA and 19 μL of RNase-free water. This was followed by an activation step at 98 °C for 30 s. Thirty amplification cycles were then carried out (98 °C for 10 s, 55 °C for 20 s, and 72 °C for 2 min), followed by a terminal extension step at 72 °C for 10 min. The PCR product was subjected to nucleotide sequencing (Comate Bioscience Co., Ltd).

### Growth curves of rescued BTV on BHK-21 cells

BHK-21 cell monolayers were cultured to confluency in 6-well tissue culture plates, and the cells were infected with wild-type BTV-1, rescued BTV-1 and cross-serotype reassortant BTV-1 at 100 TCID_50_/well, respectively. After incfection for 2 h at 37 °C, the medium was removed and refreshed with 2 mL of minimum essential medium and incubation was continued. At 24, 48, 72, 96 and 120 h post infection, 100 μL of supernatant was removed and replaced with 100 μL of fresh growth medium. The virus titers of supernatant samples were determined by serial dilution and TCID_50_ assay on BHK-21 cells.

## Results

### Preparation of helper plasmids and T7 RNA polymerase-based PCR product

Based on a previous report, helper plasmids is necessary for rescue of BTV using RG system, making the system much more versatile and generally applicable [16]. With consideration that the VP7 stabilizes the BTV replication complex, seven helper plasmids were applied in this RG system although VP7 plasmid can be omitted [16]. Helper plasmids were prepared according to Materials and Methods, and the concentration of each plasmid was adjusted to 100 ng/μL with OPTI-MEM. It has been demonstrated previously that PCR products under the control of RNA polymerase I promoter could be used to recover influenza virus [17]. These data suggested that the PCR-based RG system for BTV would be feasible since the transcripts produced from the PCR products mimicked the viral RNAs. To this end, a RG system was designed with BSR cell line and T7 polymerase promoter. As described under Materials and Methods, each PCR amplicon contained a T7 polymerase promoter and a BTV gene segment (Fig. 1). Ten segments of BTV-1(SZ97/1 strain) and segment 2 of BTV-2IT(L) were prepared as mentioned in Materials and Methods. These PCR products were purified with PCR clean Kit according to the instruction manual, and the nucleic acid concentration of each one was detected using Micro UV spectrophotometer. Then, the concentration of each PCR amplicon was adjusted to 100 ng/μL.

### Generation of viable BTV-1 from PCR product and assistant plasmids

**Fig. 3:**
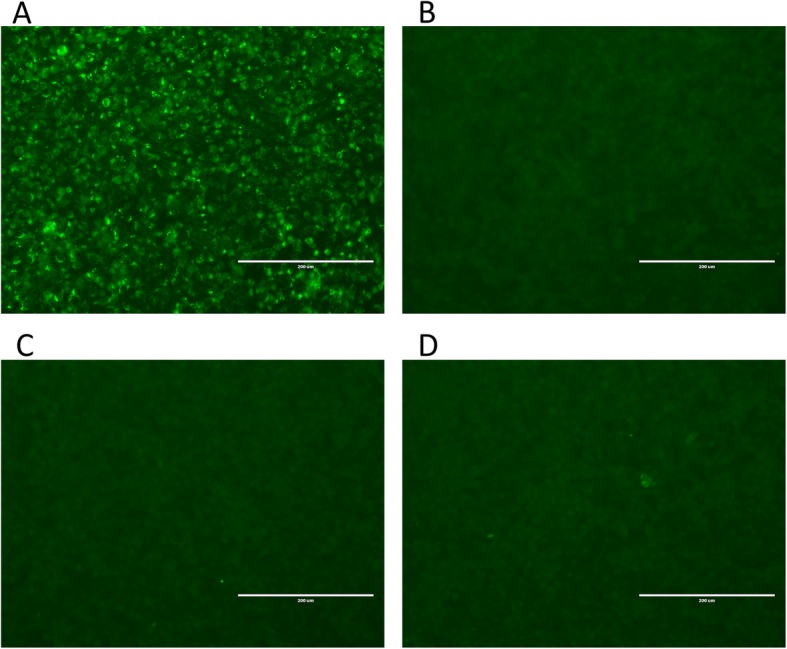
IFA was employed to confirm the recovery of infectious BTV-1 (strain SZ97/1) by transfection of BSR cells with 10 PCR amplicons and helper plasmids. The lysates from the transfected cell, mock-transfected cell, helper plasmids only transfection cell and PCR products only transfection cell were harvested and infected in BHK-21 cells; 4 days post infection, IFA was performed with anti-BTV VP7 monoclonal antibody (named BTV-4H7) and goat anti-mouse IgG FITC labelled antibody. If BTV were recovered successfully, the BHK-21 cell was further infected, and the green florescence signal could be detected with IFA. **a** Green fluorescence signal was detected in BHK-21 cells which were infected with experimental group. **b** No fluorescence was observed in BHK-21 cells infected with mock-transfected cells lysate. **c** No fluorescence was observed in BHK-21 cells infected with helper plasmids only transfected cells lysate. **d** No fluorescence was observed in BHK-21 cells infected with PCR products only transfected cells lysate

In order to determine whether infectious BTV could be rescued with the PCR-based RG system, helper plasmids and PCR products were transfected with a two-step transfection protocol as mention above. In contrast to the control groups, CPE appeared in experimental group at 4 days post transfection (data not shown). The cells from experiment group and control groups were lysed, and the supernatant was extracted. BHK-21 cells were infected with these supernatants. Then indirect immunofluorescence (IFA) assays were performed. The first antibody is the BTV group-specific Mab (BTV-4H7), this Mab showed specific reaction with BTV. If the virus was recovered successfully, this Mab will bind to VP7 of BTV and green fluorescence signal should be detected with IFA. In contrast to the control group samples, green fluorescence signal was detected from cells which were infected with sample that was transfected with helper plasmids and PCR products (Fig. 3).

### Generation of reassortant viruses

**Fig. 4:**
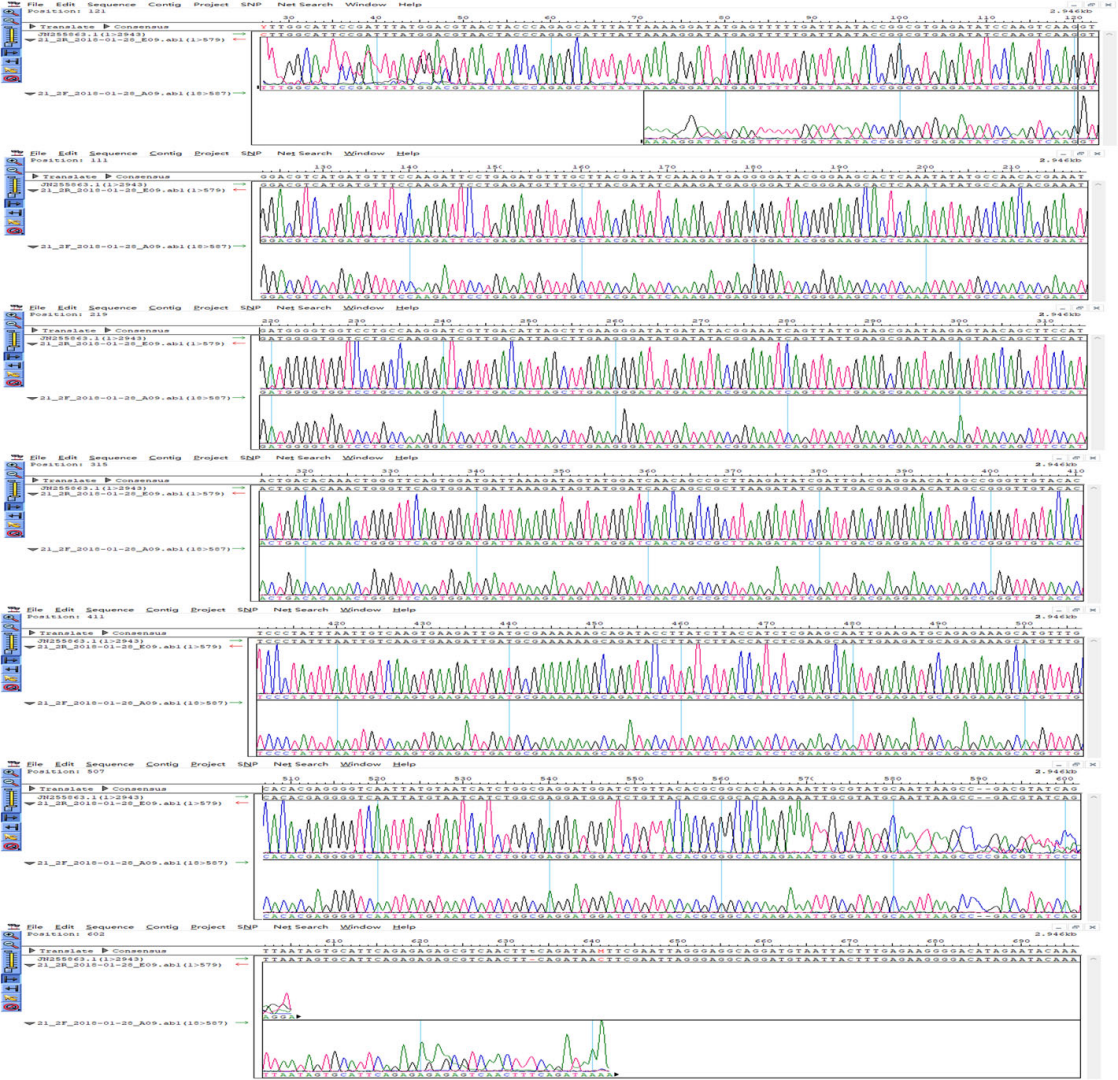
Identification of BTV1_seg2_ with part segment 2 gene sequencing. The recovery BTV dsRNA was extracted and amplified with RT-PCR by the BTV-2IT(L) segment 2 specific primers. The PCR amplicon was sequenced and aligned with segment 2 of BTV2IT(L) (JN255863), and the aligned results showed that the PCR amplicon was a part of this BTV2 segment 2. These results indicated that reassortant BTV1, which exchanged segment 2 with segment 2 of BTV2IT(L) (JN255863), was successfully rescued

One of main aims of the RG system is to produce virus with genome segment exchanged with another serotype virus. BTV segment 2 gene encodes for the outer protein VP2, this protein is related to BTV infection [9]. To further evaluate the PCR-based RG system and to determine whether this system can sufficiently rescue the recombinant virus with exchanged segment 2 of BTV-1 (Strain SZ97/1) with BTV-2IT(L), the BSR cells were transfected with helper plasmids and 10 PCR amplicons composed of the segment 2 of BTV-2IT(L) and other segments of BTV1 (strain SZ97/1) according to the method mentioned above. Reassortant virus were rescued successfully and then infected BHK-21 cell. The S2 segment-specific primer pairs for BTV-2IT(L) were used to amplify part of BTV2 segment 2 gene from extracted reassortant virus RNA. Further, sequencing was used to confirm the segment 2 gene originating from BTV-2IT(L) (Fig. 4). In fact, these specific primers targeting segment 2 of BTV-2IT(L) could not amplify segment 2 of BTV1(strain SZ97/1), the successful amplification with these primers preliminarily verified segment 2 of BTV1 (strain SZ97/1) has been exchanged with segment 2 of BTV-2IT(L). To further identify this, the amplicon sequencing was employed. By sequence alignment, the amplicon was confirmed as part of segment 2 of BTV-2IT(L) and it was 100% identical with the corresponding part. All these results were evidences that the reassortant BTV1 containing the segment 2 of BTV2IT(L) (BTV1_seg2_) was successfully rescued.

### Growth properties of wild-type and recombinant viruses in mammalian cells

**Fig. 5:**
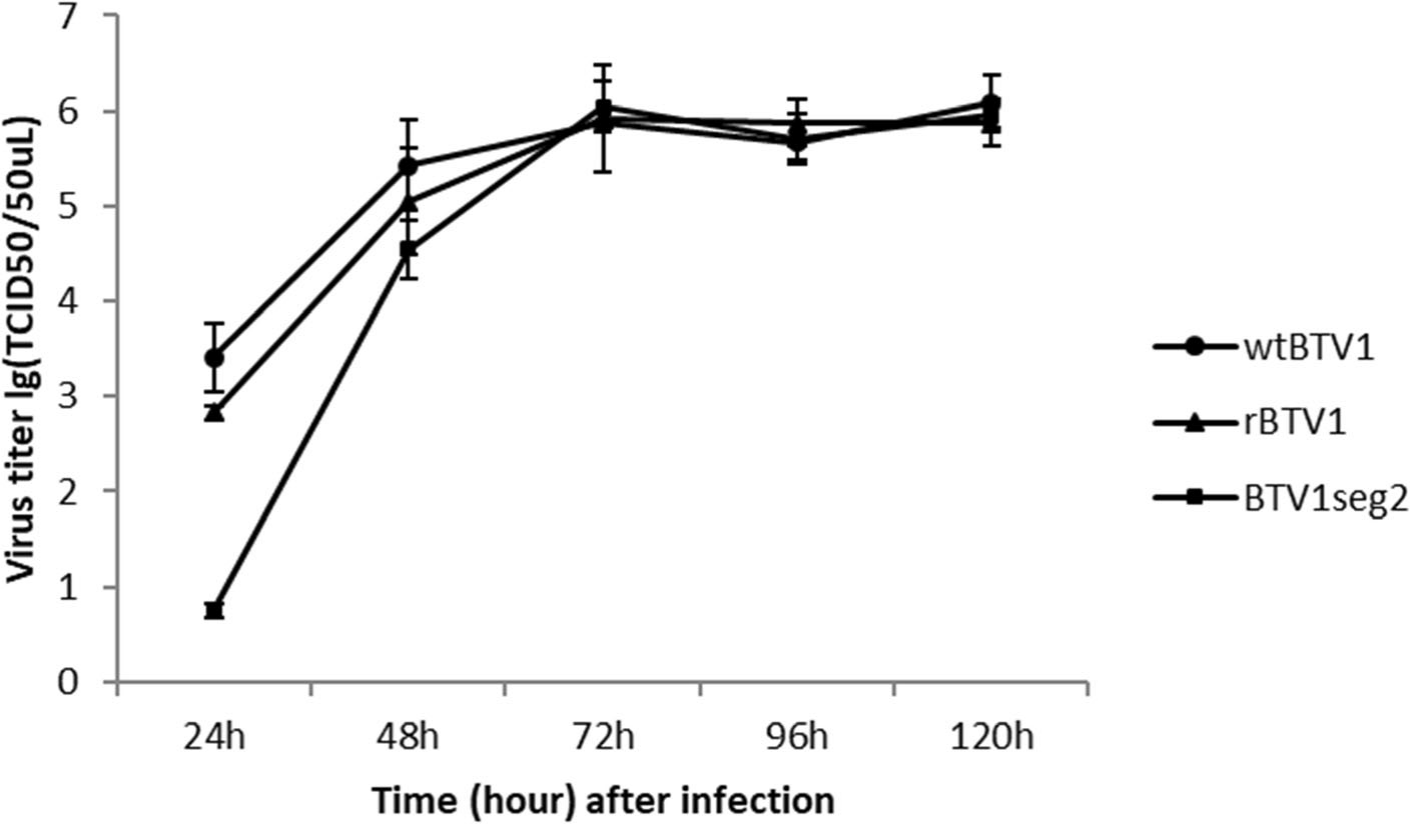
Growth curves of wild-type and recombinant viruses in BHK-21 cell. BHK-21 cells were infected with wtBTV1, rBTV1 and BTV1_seg2_, virus titers in the culture supernatant were determined at various time points post-infection. Results are presented as the mean viral titers of three independent experiments and error bars indicate the standard deviation. The viral titer of BTV1_seg2_ was lower than that of wtBTV1 and rBTV1 at 24 h and 48 h post infection, and then the titers were same at 72 h, 96 h and 120 h post infection. The titers of wtBTV1, rBTV1 and BTV1_seg2_ increased quickly within the 48 h post infection, and then the titer of each virus reach plateaus at 72 h post infection

The replicative capacities of wild-type BTV-1 (wtBTV1), rescued BTV-1 (rBTV1) and the BTV1_seg2_ were evaluated in BHK-21 cells. Cells were infected with 100 TCID_50_ of virus, and virus titers in the culture supernatant were determined at various time points post-infection. rBTV1 showed no significant difference in growth kinetics compared to that of wtBTV1. BTV1_seg2_ grew slightly slower than wtBV1 and rBTV1 for the first 48 h, however, the replication trend reached the same level of wtBV1 and rBTV1 at 72 h, 96 h and 120 h time points (Fig. 5). It seemed that the replication ability of reassortant BTV1 with exogenous segment 2 gene was weaker than the original virus, but the plateaus of the growth curves of these viruses were almost same in the platform period. It is reported that the VP2 protein surrounded by the VP5 protein binds to the surface glycoproteins of the host cell, thus facilitating endocytosis of the virion [9]. Both the binding and assembly steps of the virus are associated with the segment 2 gene of the BTV [9]. Moreover, the replication characteristic of BTV can be affected by a mutation in the segment 2, although this is not the situation for all the cases [13, 18]. This may be the reason for a difference in growth properties among these viruses.

## Discussion

Till date, strategies for rescuing BTV has mainly depended on transfecting in vitro-synthesized T7 polymerase transcripts with/without assistant plasmids, and could be named as RNA-based RG system [10–12, 19]. A complete plasmid depended RG system, thus named as plasmid-based RG system, has been reported since 2015 [13]. Both of these reported methods to recover infectious BTV need many plasmid constructions which further requires insertion of BTV gene into special vector. The plasmid that could be used as template for RNA synthesis in RNA-based RG system should introduce a T7 polymerase promotor and a type III restriction endonuclease, and for plasmid-based RG system, not only a T7 polymerase promoter should be introduced, but also a specific hammerhead ribozyme sequence and T7 terminator should be introduced into the specific vector [13, 20]. It is not unusual to encounter cloning difficulties for some BTV genes when we insert them into specific vector. In this report, a significant modification is introduced into BTV RG system based on the PCR amplicons. For BTV RG system, the helper plasmids could improve the rescue success rate, and these plasmids could be used to recover different reassortant bluetongue viruses [16]. This indicated that if the helper plasmids were constructed, new plasmid construction is not necessary for our PCR-based RG system. There is only a need to introduce a T7 polymerase promoter, which is only 17 bp long, ahead of 5′ terminal of each BTV segment with this PCR method. Indeed, the construction of seven helper plasmids was also necessary in our RG system to improve the rescue efficiency, but as mentioned above, usually this is an “once for all job” in BTV rescue strategy in ours and in most other cases. Compared with other BTV RG systems, our method is easier to operate and time-saving, as our strategy does not need the complex RNA preparation and reduces plasmid construction steps. Hence, this is very convenient when a series of mutant virus need to be recovered.

For the PCR amplicon in this RG system, the first nucleotide of each BTV segment is located at the transcriptional start site of T7 polymerase promoter, and the end point of the amplicon is the last nucleotide of the BTV segment. Extra sequence can exist ahead of 5′ terminal of T7 polymerase promoter without any effect on transcription reaction [21], this makes primers selection easier when there is a need to prepare BTV PCR amplicons with some modified plasmids. But to the 3′ end of the amplicon, no extra gene should exist, because the T7 run-off strategy was employed. Although it has been reported that T7 RNA polymerase introduces considerable heterogeneity on the 3′ end of the transcripts, which includes premature termination of transcription and addition of a few nucleotides [21, 22]. In our RG system, run-off strategy worked very well. We could obtain the appropriate T7 run-off transcript containing the 3′ end and these transcripts were enough for the virus recovery in the PCR-based RG system.

In our RG system, the PCR amplicons preparation of each BTV segment is the most important step. In this study, the templates for PCR amplification were T-vector containing targeting gene. However, if the quantity and quality of amplicons can be guaranteed, other templates also can be applied, including different plasmids, cDNA from BTV dsRNA and synthesized genes. No matter what kind of template is applied, mutation introduced during PCR amplification must be avoided as far as possible. And this is indeed a potential problem that should be focused on and avoided even though we have not faced this issue when rescuing BTV. In fact, due to the development of high-fidelity PCR enzymes and PCR product sequencing technology, it is easier than before to get a specific gene segment without any unexcepted mutation. Based on the above reasons, high fidelity DNA polymerase is strongly recommended in our PCR-based RG method.

This RG system is highly dependent on the T7 polymerase expression level in BSR cells, so the purity of BSR cells were very important when we recovered the viruses. To maintain the purity of BSR cells, we recommend treatment of BSR cells with G418 reagent before rescuing the virus, as stably expressing cell line is easy to degenerate. Because one set of the helper plasmids is suitable for different recombinant BTV recovery in most cases and replacement of assistant plasmids with PCR products is not necessary. In other words, utilization of helper plasmids should be more convenient in BTV RG system. So, we did not try to replace helper plasmids with PCR amplicons, as reported in the influenza virus rescue study [17].

Till date, it is still not clear as to which gene or genes critically contribute to BTV pathogenicity, several mutant viruses with mutations in one or more genes could be produced to explore this scientific question using our BTV RG system. Using a combination of PCR amplicons and helper plasmids, it could be possible to streamline the study of many gene variants for one or more gene segments and determine fitness, pathogenesis or any other biological aspects of the virus. Several mutant viruses with mutations in one or more genes could be produced without RNA synthesis or with less number of plasmid constructions. This method could offer a useful reference to other similar viruses with genomes larger than the BTV or a gene difficult to construct into plasmid. In summary, an RG system for BTV was developed that does not require RNA preparation step and reduce the number of plasmids construction for the recovery of viruses, and this has profound implications for vaccine development, pandemic preparedness, and for the study of BTV.

## Conclusions

The current study has developed a PCR based RG method, this strategy is an alternative to the RNA-based and plasmid based-RG system for recovery of the BTV. We further establish this method as more convenient when compared to other available methods. Furthermore, this RG strategy is likely applicable for other Orbiviruses.

## Data Availability

Not applicable. All relevant data are within the paper.

## Abbreviations

BTV: Bluetongue virus
BTV1_seg2_: Reassortant BTV-1 contain segment 2 of BTV2IT(L)
CPE: Cytopathic effect
dsRNA: Double-stranded RNA
FITC: Fluorescein isothiocyanate
IFA: Indirect immunofluorescence
PBS: Phosphate buffered saline
PBST: Phosphate buffered saline with 0.05% Tween-20
rBTV1: rescued BTV-1
RG: Reverse genetics
TCID_50_: Tissue culture infective dose
wtBTV1: wild-type BTV-1

## References

[1] Mellor PS (1990). The replication of bluetongue virus in Culicoides vectors. Curr Top Microbiol Immunol.

[2] Gibbs EP, Greiner EC (1994). The epidemiology of bluetongue. Comp Immunol Microbiol Infect Dis.

[3] van der Sluijs MT, de Smit AJ, Moormann RJ (2016). Vector independent transmission of the vector-borne bluetongue virus. Crit Rev Microbiol.

[4] Mayo C (2016). A deterministic model to quantify risk and guide mitigation strategies to reduce bluetongue virus transmission in California dairy cattle. PLoS One.

[5] Toussaint J (2006). Bluetongue in northern Europe. Vet Rec.

[6] Jenckel M, et al. Complete coding genome sequence of putative novel bluetongue virus serotype 27. Genome Announc. 2015;3(2):e00016-15. 10.1128/genomeA.00016-15 PMC4357740 25767218

[7] Verwoerd DW, Louw H, Oellermann RA (1970). Characterization of bluetongue virus ribonucleic acid. J Virol.

[8] Ratinier M (2016). Bluetongue virus NS4 protein is an interferon antagonist and a determinant of virus virulence. J Virol.

[9] Roy P (2017). Bluetongue virus structure and assembly. Curr Opin Virol.

[10] Boyce M, Roy P (2007). Recovery of infectious bluetongue virus from RNA. J Virol.

[11] Boyce M, Celma CC, Roy P (2008). Development of reverse genetics systems for bluetongue virus: recovery of infectious virus from synthetic RNA transcripts. J Virol.

[12] Matsuo E, Roy P (2009). Bluetongue virus VP6 acts early in the replication cycle and can form the basis of chimeric virus formation. J Virol.

[13] Pretorius JM, Huismans H, Theron J (2015). Establishment of an entirely plasmid-based reverse genetics system for bluetongue virus. Virology.

[14] Yang T (2014). Establishment of a reverse genetics system for bluetongue virus. Chin J Prev Vet Med.

[15] Xu Q (2015). Isolation of a bluetongue virus group-specific monoclonal antibody and application to a diagnostic competitive ELISA. Appl Microbiol Biotechnol.

[16] Van Rijn PA (2016). Requirements and comparative analysis of reverse genetics for bluetongue virus (BTV) and African horse sickness virus (AHSV). Virol J.

[17] Chen H (2012). Partial and full PCR-based reverse genetics strategy for influenza viruses. PLoS One.

[18] Feenstra F, Pap JS, van Rijn PA (2015). Application of bluetongue disabled infectious single animal (DISA) vaccine for different serotypes by VP2 exchange or incorporation of chimeric VP2. Vaccine.

[19] Matsuo E, Roy P (2013). Minimum requirements for bluetongue virus primary replication in vivo. J Virol.

[20] Matsuo E (2011). Generation of replication-defective virus-based vaccines that confer full protection in sheep against virulent bluetongue virus challenge. J Virol.

[21] Milligan JF (1987). Oligoribonucleotide synthesis using T7 RNA polymerase and synthetic DNA templates. Nucleic Acids Res.

[22] Martin CT, Muller DK, Coleman JE (1988). Processivity in early stages of transcription by T7 RNA polymerase. Biochemistry.

